# Correlation between arterial lactate and venous lactate in patients with sepsis and septic shock

**DOI:** 10.1186/cc13367

**Published:** 2014-03-17

**Authors:** P Theerawit, C Na Petvicham

**Affiliations:** 1 Ramathibodi Hospital, Mahidol University, Bangkok, Thailand

## Introduction

Measurement of arterial lactate (A-LACT) levels has been used to monitor poor tissue perfusion, predicting mortality and guiding resuscitation. Peripheral venous lactate (V-LACT) has been regarded as an unreliable test, but a less invasive approach. We aimed to determine correlation between A-LACT and V-LACT and agreement of both in order to determine the usefulness of V-LACT as a biomarker for assessment in sepsis.

## Methods

We conduct a prospective, cross-sectional study during June to December 2011 at a university hospital. Septic patients in the ICU were enrolled in this research. Sepsis was defined according to the Surviving Sepsis Campaign: International Guidelines for Management of Severe Sepsis and Septic Shock: 2008. The exclusion criteria were: contraindication for arterial puncture; and denying inform consent. The venous lactate would be sampled at the same point in time as arterial lactate measurement. The correlation and agreement between arterial and venous lactate was the primary outcome.

## Results

**Figure 1:**
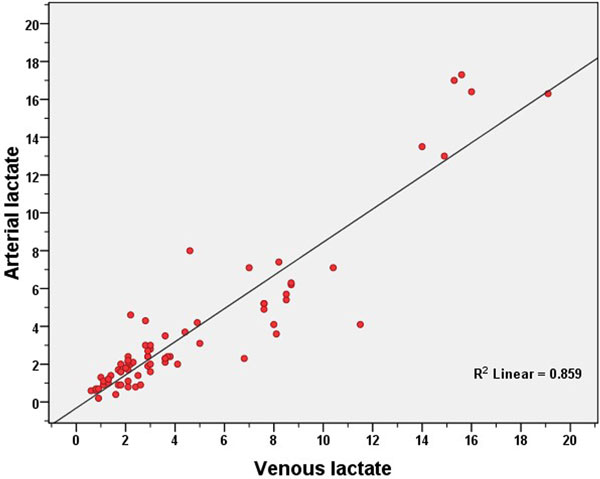


**Figure 2:**
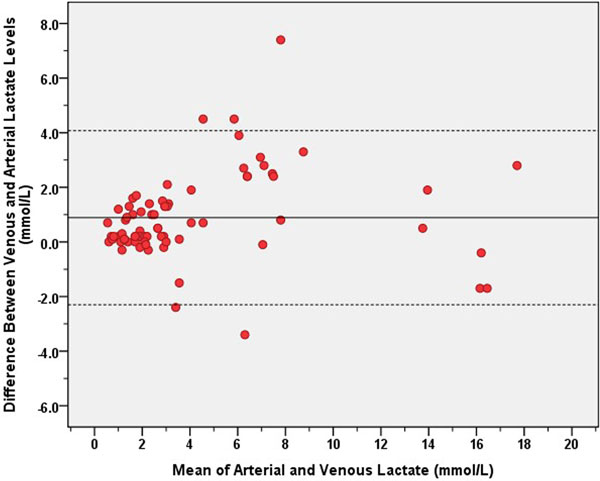


A total of 73 pair-samples in 45 intensive care patients were collected. Mean age was 68.33 ± 14.5 years. Fifty percent of all patients received the vasopressors to stabilize hemodynamics. The mean serum creatinine level was 2.78 mg/dl and the mean anion gap was 13.55 mmol/l. The mean arterial lactate (A-LACT) level was 3.73 ± 4.0 mmol/l, and the mean venous lactate (V-LACT) level was 4.6 ± 4.2 mmol/l. The A-LACT and V-LACT were strongly correlated as shown in Figure 1 (*r *= 0.927, *P *< 0.0001, *r*^2 ^= 0.859). The mean difference between V-LACT and A-LACT was 0.889 mmol/l. The 95% limits of the V-A difference in the individual patients were between −2.3 and 4.1 mmol/l. However, the agreement looks very good at lactate levels not higher than 4 mmol/l (Figure 2). The regression equation was: A-LACT = (0.877 × V-LACT) - 0.320.

## Conclusion

The arterial lactate and venous lactate levels were strongly correlated in the condition of sepsis or septic shock. Consequently, V-LACT may be used in substitution for A-LACT particularly in lactate levels not higher than 4 mmol/l. However, trending should be generally applied instead of the absolute value.

